# Technical Note on Vestibuloplasty around Dental Implants Using Erbium YAG Laser-Assisted Periosteal Fenestration (LA-PF)

**DOI:** 10.3390/medicina59101884

**Published:** 2023-10-23

**Authors:** Kyeong-Ok Lim, Won-Pyo Lee

**Affiliations:** Department of Periodontology, School of Dentistry, Chosun University, Gwangju 61452, Republic of Korea; periolim90@chosun.ac.kr

**Keywords:** dental implants, laser, mouth mucosa, oral surgical procedures, peri implantitis, solid state, vestibuloplasty

## Abstract

Various vestibuloplasty techniques have been reported to increase the attached mucosa (AM) and vestibular depth around dental implants. However, these surgical methods have disadvantages, such as limitations in manipulation, necessity of suturing, postoperative discomfort, swelling, and pain. This study aimed to evaluate the efficacy of laser-assisted periosteal fenestration (LA-PF) in treating patients with a shallow vestibule and insufficient AM around dental implants. LA-PF was performed using an Erbium YAG laser (Er:YAG laser). First, a partial-thickness, apically positioned flap was used. A horizontal periosteal fenestration was performed using an Er:YAG laser to expose the bones. Periosteal suturing was not required. After 12 months, sufficient AM and deep vestibules were obtained and maintained. Thus, the LA-PF technique may be a simple and predictable treatment modality for shallow vestibules with insufficient AM around dental implants.

## 1. Introduction

The need for attached mucosa (AM) for peri-implant health and long-term stability is still being researched [1]. Lin et al. reported that a sufficient amount of AM around dental implants is associated with less plaque accumulation and a low incidence of peri-implantitis [2]. Tavelli et al. reported that implants with an AM < 2 mm could lead to increased patient discomfort, loss of marginal bone, and increased bleeding during probing [3]. Monje and Blasi reported that a minimum AM of 2 mm was even more important, especially for irregular maintenance compliance, as it acts as a protective factor against peri-implantitis [4]. Thus, the creation of an immobile AM of at least 2 mm is important for long-term peri-implant health [5].

Khoury et al. reported that an apically positioned flap (APF) combined with a free gingival graft (FGG) is the best treatment modality to increase the AM [6]. However, FGG requires the formation of a donor site, which increases patient discomfort, surgical time, and the risk of postoperative complications [7]. APF is a relatively simple technique but has the disadvantage of a high risk of relapse [8].

To compensate for these disadvantages, we introduced modified periosteal fenestration (mPF) in a previous study to form a sufficient AM and vestibule around the implant [9]. Unlike FGG, mPF did not form a donor site but instead compensated for the high recurrence tendency of APF through periosteal fenestration. However, a skilled technique is required to perform periosteal suturing. Subsequently, in a follow-up study, we introduced simplified periosteal fenestration, which makes periosteal suturing easier [10]. However, as both techniques involve surgery using a scalpel, securing the field of vision is difficult, owing to bleeding during the partial-thickness flap. Manipulation and periosteal suturing may not be possible in posterior molar regions.

In this case report, we describe the technical aspects of laser-assisted periosteal fenestration (LA-PF) using an Erbium YAG (Er:YAG) laser, which makes it easier to form an AM around a dental implant without forming a donor site or periosteal suturing.

## 2. Case Report

### 2.1. Surgical Procedures of Er:YAG Laser-Assisted Periosteal Fenestration (LA-PF)

All procedures and measurements related to the LA-PF technique were performed by a proficient periodontologist (W.-P.L.). The Er:YAG laser system employed for these procedures was the LiteTouch™ model (Shinhung Co., Seoul, Republic of Korea). The laser system was equipped with both contact and noncontact handpieces, and the laser settings were configured with a pulse energy of 200 mJ and a frequency of 18 Hz, complemented by a continuous water spray mechanism. The initial step involved the creation of a partial-thickness flap positioned precisely 2–3 mm below the margin of the peri-implant soft tissue, which is an essential aspect of the LA-PF technique. The procedure was performed under local anesthesia to ensure patient comfort and cooperation. The flap was meticulously fashioned to reach the depth at which vestibule formation was required. The inherent hemostatic properties of the Er:YAG laser proved invaluable in minimizing intraoperative bleeding and facilitating an unobstructed field of vision throughout the surgical process. Notably, the efficacy of the Er:YAG laser eliminated the need for periosteal sutures to achieve hemostasis. After flap creation, periosteal fenestration was meticulously performed. Using the Er:YAG laser, a band-shaped exposure of the alveolar bone, approximately 2 mm in width, was precisely achieved in the apical region of the divided lower flap. To protect the surgical wound and aid in its healing process, an absorbable periodontal dressing (Reso-Pac^®^, Hager & Werken GmbH & Co. KG, Duisburg, Germany) was meticulously applied. To manage postoperative discomfort and promote recovery, patients received a regimen of postoperative analgesia (aceclofenac 100 mg, Dona-A ST, Seoul, Republic of Korea) twice daily for a period of 1 week. In cases where a non-absorbable periodontal dressing (Coe-Pac^TM^, GC America Inc., Lucerne, Switzerland) was used, it was typically removed approximately 1 week postoperatively to ensure an optimal healing environment and patient comfort.

### 2.2. Case

**Figure 1:**
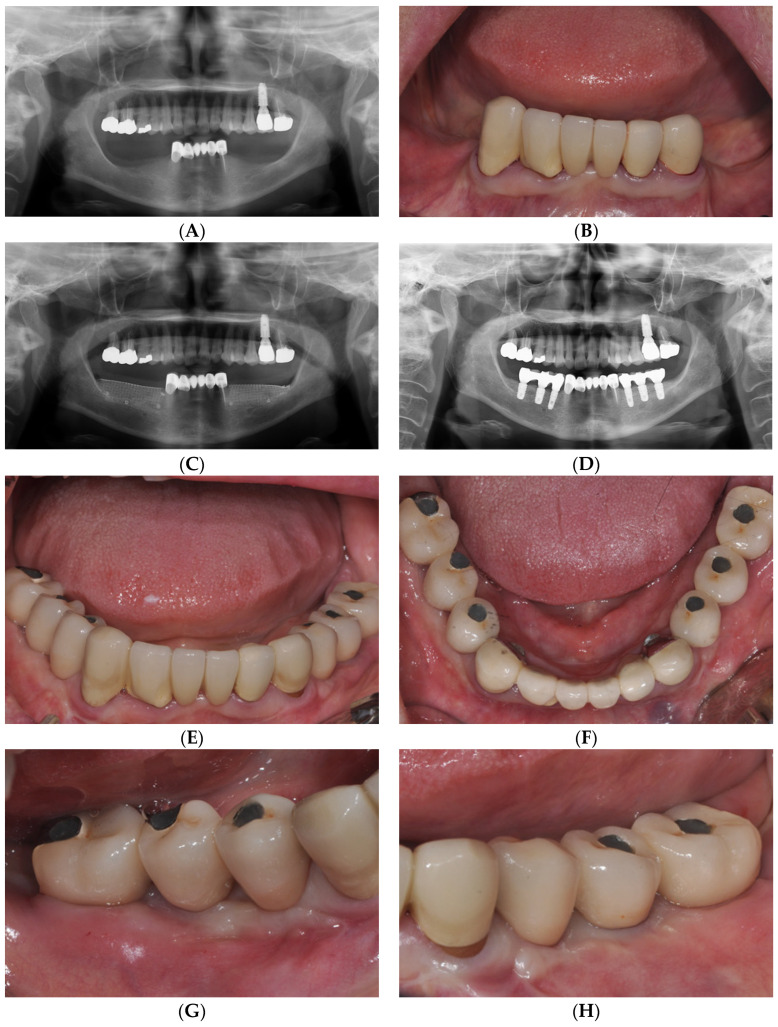
(**A**) Panoramic radiograph and (**B**) clinical condition at the initial examination. (**C**) Panoramic radiograph after (**C**) vertical ridge augmentation and (**D**) the final prosthesis. (**E**) Frontal and (**F**) occlusal view after the final prosthesis. Peri-implant soft tissue condition of (**G**) right and (**H**) left molar area after the final prosthesis.

A 65-year-old female visited a dental hospital with a desire to place implants in the posterior teeth on both sides of the mandible. This patient had been using dentures for the past 20 years. As a result, the ridges of the lower posterior teeth on both sides atrophied, and the keratinized gingiva narrowed to 1–2 mm in width. For implant placement, vertical and horizontal guided bone regeneration was first performed using an allograft (Do Bone^®^, CGBio, Seongnam, Republic of Korea) and titanium mesh (Ti-mesh). After 5 months, the Ti-mesh was removed, and the implants were placed. After prosthetic fabrication, insufficient AM was observed on the buccal sides of the implants. In particular, the vestibule became shallow toward the posterior molars. This may be the result of vertically guided bone regeneration along with insufficiently keratinized gingiva (Figure 1).

**Figure 2:**
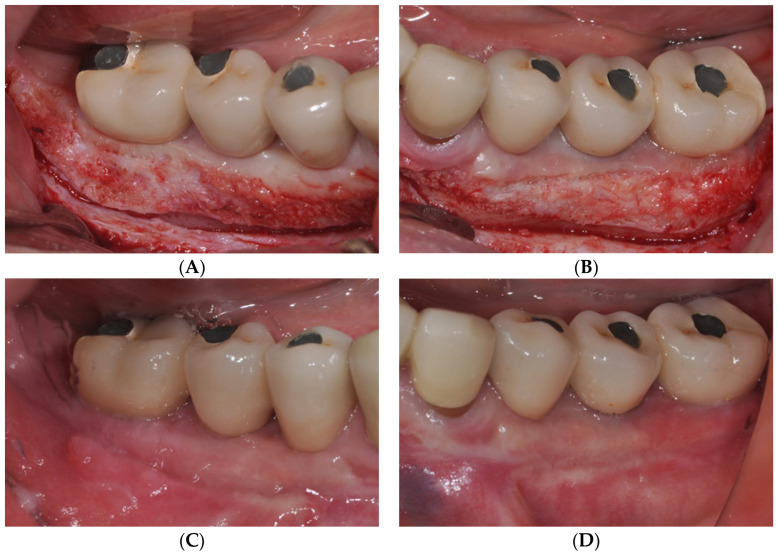
(**A**,**B**) Buccal view immediately after performing laser-assisted periosteal fenestration (LA-PF). (**C**,**D**) Clinical view at 12 months after LA-PF. Increased AM was observed around the implants.

Therefore, the aforementioned LA-PF technique was used to increase the depth of the AM and vestibule. An APF of approximately 6 mm was observed immediately postoperatively. At 12 months postoperatively, a favorable AM and a vestibular depth of approximately 4 mm were maintained at most implant sites (Figure 2).

## 3. Discussion

In this case, we determined whether the LA-PF could effectively form an immobile AM and vestibule around the dental implants. Unlike other vestibuloplasty procedures, the LA-PF technique does not require periosteal sutures or donor sites, which simplifies the surgery and reduces the procedure time. Nevertheless, the LA-PF procedure achieved and maintained an AM of approximately 4 mm at the 12-month follow-up period, similar to traditional surgical techniques, such as FGG. 

APF combined with FGG is the most effective method for acquiring AM [4,7]. However, our previous study showed that periosteal fenestration could replace the FGG as an effective vestibuloplasty technique [11]. This study compared changes in AM width at 1 year after vestibuloplasty using APF, FGG, and mPF around mandibular posterior implants. All three procedures required an AM of approximately 6 mm immediately after surgery. However, a tendency for recurrence existed, approximately 2 mm for APF and 4 mm for FGG and mPF, at 1 year postoperatively. Periosteal fenestration has a lower tendency of recurrence than APF alone, because the alveolar bone exposed after periosteal fenestration undergoes secondary healing as a band of scar tissue. Kon et al. explained this clinically and histologically using animal experiments and reported that collagen fibrils were embedded at a site where the exposed alveolar bone was resorbed to form a scar tissue band [12]. This scar tissue band acts as a physical barrier that reduces the tendency of the repositioned buccal muscle to return to its original position. Thus, periosteal fenestration is less prone to recurrence than APF and can attain AM as predictable as FGG [13,14]. 

**Figure 3:**
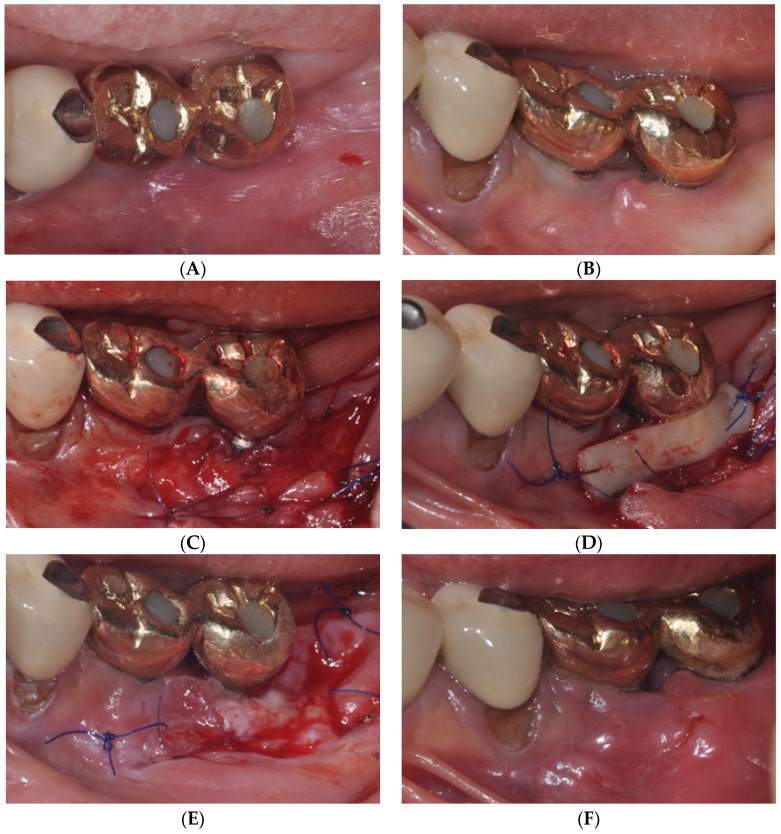
A 65-year-old woman with deficient attached mucosa (AM) in #36, 37 implants (**A**) Occlusal and (**B**) buccal view at the initial examination. (**C**) Buccal view immediately after performing partial-thickness, apically positioned flap (APF). (**D**) Buccal view immediately after free gingival graft (FGG). (**E**) Healing condition at 1 week postoperatively. (**F**) Clinical view of #36, 37 implants at 4.5 months after FGG. Postoperative relapse was observed.

**Figure 4:**
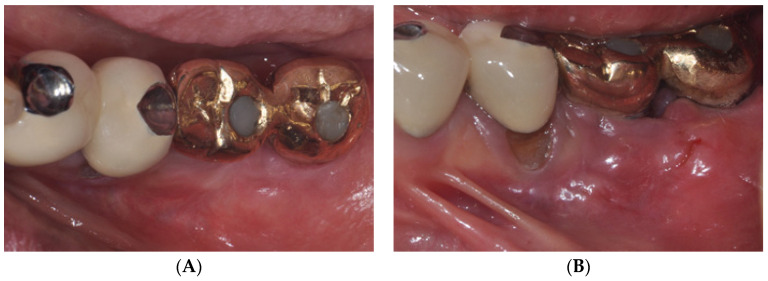

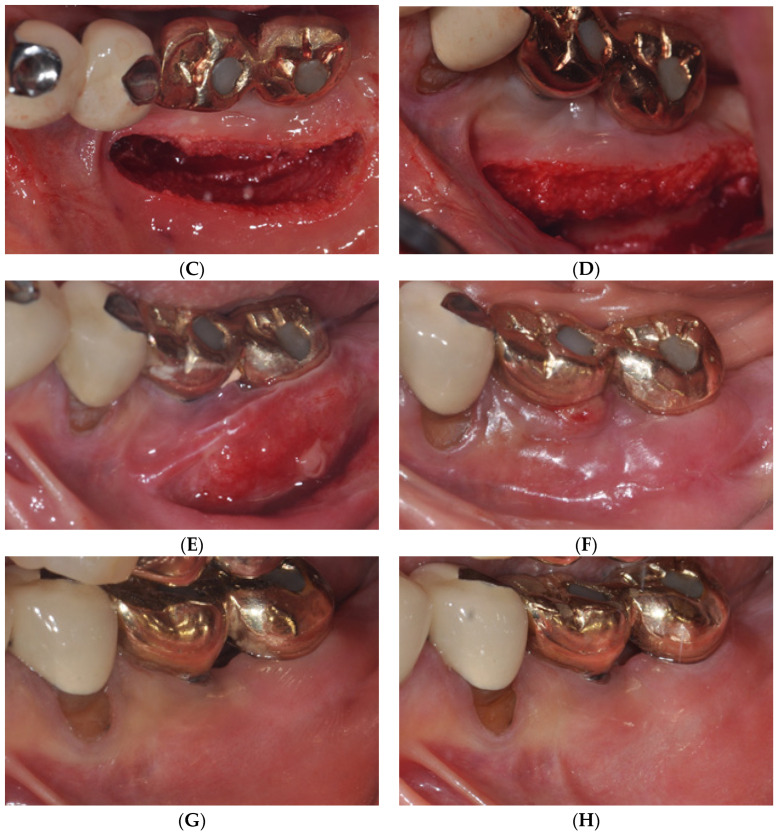
The laser-assisted periosteal fenestration (LA-PF) technique was performed in the same patient as in Figure 3. (**A**) Occlusal and (**B**) buccal view at the initial examination. (**C**) Occlusal and (**D**) buccal view immediately after performing LA-PF. (**E**) Healing condition at 1 week postoperatively. (**F**) Clinical view at 2 months postoperatively. (**G**) Clinical view of #36, 37 implants at 1 year after LA-PF. (**H**) Clinical view of #36, 37 implants at 3 years after LA-PF. Increased attached mucosa remained stable for 3 years postoperatively.

FGG requires a high level of surgical skill to form the recipient and donor sites. In other words, when the fixation of the free gingiva is insufficient or excessive mobile tissue is present at the recipient site, the prognosis of FGG becomes poor (Figure 3). In contrast, LA-PF, in addition to the advantage of not requiring a donor site, does not require a high level of surgical skill because it does not require proper recipient site formation or graft stability, which are required for the success of FGG (Figure 4).

Conventional surgical vestibuloplasty techniques can cause bleeding, limited manipulation, postoperative pain, and swelling, which reduce patient satisfaction [15]. The use of lasers in dental procedures offers several advantages. Laser therapy provides a hemostatic effect that enhances visibility, streamlines surgical steps, and reduces treatment duration [16,17]. Although its inception in medical applications dates back to the mid-20th century, laser therapy emerged as a cornerstone of routine dental practice, with widespread adoption in the 1980s [18]. Lasers can be categorized into solid-state (e.g., Nd:YAG, Er:YAG, and ErCr:YSGG), gas (e.g., CO_2_), and semiconductor (diode) lasers. Laser applications are diverse, with some being effective for both soft and hard tissues (e.g., Nd:YAG, Er:YAG, ErCr:YSGG), whereas others are primarily used in soft tissues (e.g., CO_2_ and diode lasers) [19,20]. In the 1970s, CO_2_ lasers marked the first foray in oral surgery for soft tissues. Subsequently, lasers, such as diodes, Nd: YAG, and Er:YAG, have been used in periodontics, implantology, orthodontics, and restorative dentistry, encompassing both hard (bone, enamel, and dentin) and soft tissues [21]. Notably, the Er:YAG laser operates at a wavelength of 2940 nm and exhibits particular affinity for hydroxyapatite. This attribute makes it a favored choice for the treatment of hard dental tissue [22]. Moreover, employing an Er:YAG laser on soft tissue yields several advantages in routine dentistry, including reduced anesthesia requirements, fewer sutures, diminished postoperative sensitivity, selective removal of diseased tissue, accelerated healing, decreased swelling, and a lower risk of infection [23]. Especially, the Er:YAG laser has better absorption in water than the CO_2_ or Nd:YAG lasers. Thermal-mechanical tissue ablation can be performed in a narrow layer [24]. Therefore, a more precise incision is possible, and thermal damage to the adjacent tissues can be minimized. The carbonized tissue layer is thinner than that of other lasers, and some bleeding may be observed; however, the healing process is faster, which can reduce pain or discomfort after surgery. Compared to the diode laser, less thermal damage was reported; in particular, less osteonecrosis was observed with the use of the Er:YAG laser [25]. Valerie et al. investigated the histological effects of 32 excisional biopsies of buccal mucosal fibrous hyperplasia using CO_2_ and Er:YAG lasers, as described in detail in this study. The Er:YAG laser specimens exhibited significantly smaller areas of thermal damage compared to the histological evaluations. This indicates that tissue integrity is better preserved following Er:YAG laser excision, primarily because of the reduced thermal impact, which results in a smaller area of damage, as observed in histological examinations [26]. 

This study had certain inherent limitations associated with its case study design. Prospective clinical investigations, using robust methodologies and larger sample sizes are warranted to validate our findings. Furthermore, acknowledging that the LA-PF technique has certain drawbacks compared to traditional approaches, such as FGG, is essential. Unlike FGG, which relies on blade-based incisions, LA-PF requires laser equipment in the dental clinic. Among laser equipment options, diode lasers are relatively cost-effective, whereas the Er:YAG lasers employed in this study are generally more expensive. In addition, although the LA-PF procedure effectively enhances AM, it does not contribute to an increase in peri-implant mucosal thickness, which is a characteristic feature of FGG. Therefore, a partial-thickness flap positioned 2–3 mm below the peri-implant soft tissue margin should be created to mitigate the risk of marginal bone loss during LA-PF. Therefore, the traditional FGG method is recommended in cases where an increase in soft-tissue thickness is required in conjunction with AM enhancement. 

Moreover, additional precautions should be considered when performing LA-PF. Special attention must be paid to the risk of osteonecrosis, particularly when employing alternative laser types such as CO_2_ or diode lasers, as opposed to Er:YAG lasers in LA-PF procedures. This concern arises from the potential for excessive thermal damage during periosteal fenestration when CO_2_ or diode lasers are used in LA-PF procedures. Therefore, in the LA-PF technique, which requires periosteal fenestration to expose the alveolar bone, prioritizing the use of Er:YAG lasers over diodes and CO_2_ lasers is recommended. Furthermore, post-procedural hemostasis warrants careful attention during LA-PF procedures. Given the nature of the procedure, which results in secondary intention healing, patients taking antithrombotic medications, such as aspirin or warfarin, should either postpone the procedure or take additional precautions to manage potential bleeding risks.

## 4. Conclusions

In this case report, we suggest that LA-PF could be a simple and predictable treatment modality for vestibuloplasty and for increasing the insufficient AM around implants. In particular, the necessity for periosteal suturing, which is required in conventional techniques, has been eliminated, making it easier for clinicians to apply this technique. In addition, Er:YAG laser can be used to reduce pain and discomfort, leading to patient compliance with treatment. However, as the number of cases was limited, large-scale, long-term, and in-depth studies on LA-PF are necessary.

## Data Availability

The datasets generated or analyzed during the current study are available from the corresponding author upon reasonable request.
